# *Schisandra Chinensis* Lignans Suppresses the Production of Inflammatory Mediators Regulated by NF-κB, AP-1, and IRF3 in Lipopolysaccharide-Stimulated RAW264.7 Cells

**DOI:** 10.3390/molecules23123319

**Published:** 2018-12-14

**Authors:** Gan Luo, Brian Chi-Yan Cheng, Hui Zhao, Xiu-Qiong Fu, Ran Xie, Shuo-Feng Zhang, Si-Yuan Pan, Yi Zhang

**Affiliations:** 1 School of Chinese Materia Medica, Beijing University of Chinese Medicine, Beijing 100102, China; luna049@126.com (G.L.); shuofengzhang@sina.com (S.-F.Z.); siyuan-pan@163.com (S.-Y.P.); 2 College of Professional and Continuing Education, Hong Kong Polytechnic University, Hong Kong 999077, China; brichian@hotmail.com; 3 Quality Healthcare Medical Services, Hong Kong 999077, China; 4 School of Traditional Chinese Medicine, Capital Medical University, Beijing 100069, China; zhaohuishouyi@sina.com; 5 Centre for Cancer and Inflammation Research, School of Chinese Medicine, Hong Kong Baptist University, Hong Kong 999077, China; 13480405@life.hkbu.edu.hk; 6 Institute of Chinese Materia Medica, China Academy of Chinese Medical Sciences, Beijing 100700, China; xieran9636@163.com

**Keywords:** *Schisandra Chinensis* lignans, anti-inflammation, AP-1, NF-κB, IRF3, RAW264.7 macrophages

## Abstract

Schisandra Fructus (SF) is a traditional Chinese herb used in the treatment of inflammatory disorders like hepatitis. One of the main anti-inflammatory components of SF is the lignans. However, the underlying anti-inflammatory mechanism of *Schisandra Chinensis* lignans (SCL) remains unclear. This study aims to investigate the effects of SCL on inflammatory mediators in lipopolysaccharide-stimulated RAW264.7 cells and explore the underlying mechanism. The production of nitric oxide (NO) was determined by Griess reaction. ELISA was used to determine cytokine levels and chemokines secretion. To estimate protein levels and enzyme activities, we employed Western blotting. Nuclear localization of NF-κB, AP-1, and IRF3 was detected using immunofluorescence analyses. The results showed that SCL significantly reduced the release of inflammatory mediators, including NO and PGE2, which may be related to down-regulation of iNOS and COX-2 expression. The production of cytokines and chemokines was suppressed by SCL treatment. SCL also decreased the phosphorylation of IKKα/β, IκB-α, Akt, TBK1, ERK, p38, JNK, NF-κB (p65), AP-1 (c-Jun), and IRF3 in RAW264.7 macrophages activated with LPS. The nuclear protein levels and nuclear translocation of AP-1, NF-κB and IRF3 were suppressed by SCL. These results indicated that SCL suppressed the IKKα/β/NF-κB, MAPKs/AP-1 and TBK1/IRF3 signaling pathways in LPS-stimulated RAW264.7 macrophages.

## 1. Introduction

Inflammation is a defensive response that evolved in higher organisms to protect them from infection and injury [1]. However, excessive and continuous inflammation can lead to tissue damage or a series of diseases including hepatitis, pneumonia, rheumatoid arthritis and asthma etc. [2,3,4]. Currently, treatment options for inflammatory diseases have been limited to anti-inflammatory medications (e.g., acetaminophen) and steroid hormones [5]. However, long-term administration of these medications does not provide satisfactory efficacy and even causes serious adverse effects [6,7]. Therefore, safe and effective novel targeted therapeutic agents are urgently needed.

Macrophages are important components of the mononuclear phagocyte system, and play a critical role in initiation, maintenance, and resolution of inflammation [8]. Macrophages can be stimulated by various harmful stimuli, like lipopolysaccharide (LPS), an endotoxin that can initiate toll-like receptor (TLR)-4 signaling pathways, which activate transcription factors like nuclear factor-κB (NF-κB), activator protein-1 (AP-1) and interferon regulatory factor 3 (IRF3), leading to the release of pro-inflammatory cytokines and chemokines [9]. Overproduction of these inflammatory mediators will further activate immune cells and result in tissue damage and hemodynamic changes, organ failure, and ultimately death [10,11]. Thus, the TLR4 signaling pathway is one of the targets in treating inflammatory diseases.

Traditional Chinese Medicine (TCM) has thousands of years of history and is one of the forms of alternative medicine endorsed by World Health Organization [12]. TCM has increasingly been accepted by more and more people worldwide, especially for the management of inflammatory diseases, such as hepatitis, asthma, rheumatoid arthritis, and osteoarthritis [13,14,15]. Schisandrae Fructus (SF, 北五味子 Bei-Wu-Wei-Zi in Chinese), the dried fruit of *Schisandra Chinensis* (Turcz.) Baill, is found in northern China, Korea and adjacent areas in Russia [16], and has been used as a tonic medicine in healthcare [17]. SF contains large amounts of essential oil, tannins, resins, and staining materials [16]. It has been reported that the most active component of SF is the lignans, which has a wide spectrum of pharmacological activities including hepatoprotective, anti-inflammatory, anti-oxidant, and anti-tumour properties [18,19,20,21]. Previous studies reported that the dibenzocyclooctadiene lignans derivatives isolated from SF, such as Schisandrin A and Schisandrin B, have potent anti-inflammatory actions via suppressing the phosphorylation of MAPKs [22]. However, as the most effective part of SF, the mechanism underlying anti-inflammatory effect of *Schisandra Chinensis* lignans (SCL) remains largely unknown. Macrophages play an important role in many inflammatory diseases and RAW264.7 murine macrophage line is one of the most commonly used cell models to evaluate the anti-inflammatory effect of drugs in vitro [23]. In this study, we investigated the inhibitory effect of SCL on inflammatory mediators in LPS-stimulated RAW264.7 cells and explored the underlying mechanisms of this action.

## 2. Results

### 2.1. Characterization of SCL

**Figure 1:**
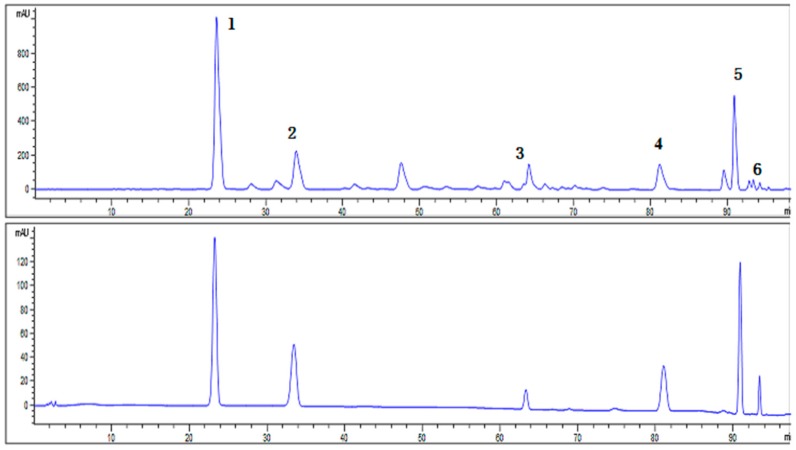
Characterization of SCL. High performance liquid chromatography chromatograms of the SCL and standards (Schisandrin, Schisandrol B, Schisantherin A, Schisandrin A, Schisandrin B and Schisandrin C) were detected at 220 nm.

The HPLC chromatogram showed that Schisandrin, Schisandrol B, Schisantherin A, Schisandrin A, Schisandrin B and Schisandrin C were present in SCL (Figure 1). The mean contents of Schisandrin, Schisandrol B, Schisantherin A, Schisandrin A, Schisandrin B and Schisandrin C per gram of SCL were 303.03 mg, 89.86 mg, 44.93 mg, 57.47 mg, 103.45 mg and 7.84 mg, respectively (Table 1).

### 2.2. SCL Decreased the Release of NO and PGE2 in RAW264.7 Cells

**Figure 2:**
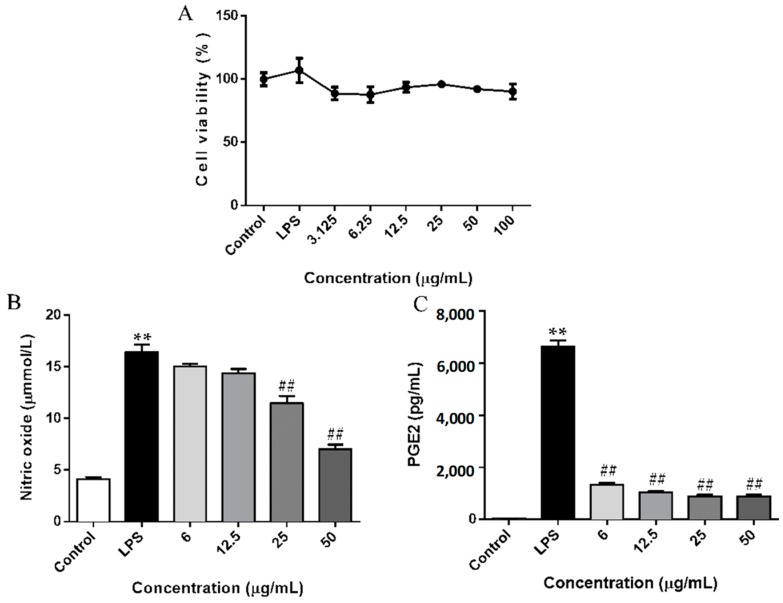
Effect of SCL on NO and PGE2 production in LPS-stimulated RAW264.7 cells. Cells were treated with SCL at various concentrations (3.125, 6.25, 12.5, 25 and 50 μg/mL) for 1 h, and then stimulated with or without LPS for 24 h, cell viability was analysed with an MTT method. (**A**). The cells were incubated with indicated concentrations of SCL for 1 h, and then stimulated with LPS for 24 h. The concentration of NO (expressed as nitrite) and PGE2 in the culture medium were quantified by Griess reaction and ELISA, respectively (**B**,**C**). The data presented in bar charts are mean ± standard error of the mean (SEM) values from 4 independent experiments. ** *p* < 0.01 vs. unstimulated cells. ^##^ *p* < 0.01 vs. LPS-stimulated cells.

To determine the sub-lethal concentrations of SCL, an MTT assay was firstly used. The viability of RAW264.7 cells was not significantly altered by 24 h incubation with up to 50 μg/mL of SCL in the presence of 1 μg/mL of LPS (Figure 2A). Therefore, the concentrations of SCL 6, 12.5, 25 and 50 μg/mL were selected in subsequent experiments. When stimulated with LPS for 24 h, the levels of nitrite (a stable oxidized product of NO) and PGE2 in the culture medium of RAW264.7 cells were significantly increased. Pre-treatment with SCL concentration dependently decreased LPS-induced NO and PGE2 production (Figure 2B,C).

### 2.3. SCL Reduced the Expression of iNOS and COX-2 in LPS-Stimulated RAW264.7 Cells

**Figure 3:**
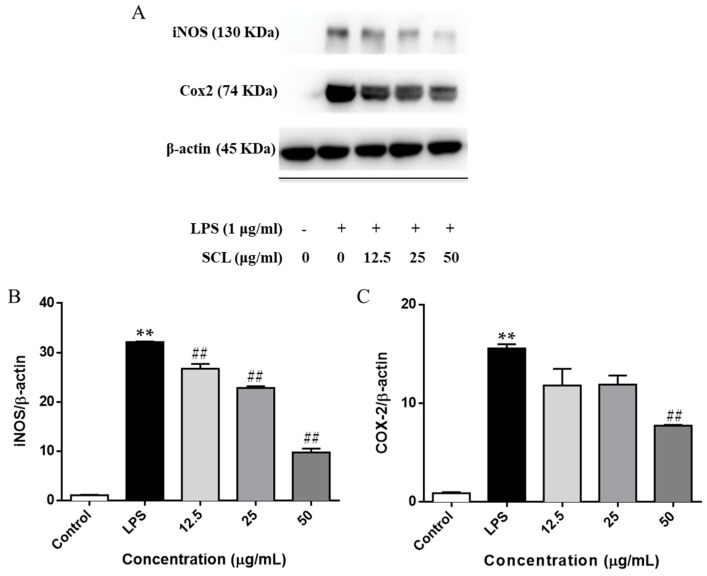
Effect of SCL on iNOS and COX-2 expression in LPS-stimulated RAW264.7 cells. Cells were treated with SCL at various concentrations (12.5, 25 and 50 μg/mL) for 1 h, and then stimulated with or without LPS for 24 h, the expression of iNOS and COX-2 were investigated by Western blotting. (**A**). The data presented in bar charts indicates the relative levels of iNOS and COX-2 (**B**,**C**). Values given are the mean ± SEM of 3 independent experiments; ** *p* < 0.01 vs. unstimulated cells. ^##^ *p* < 0.01 vs. LPS-stimulated cells.

We next investigated the protein expression of iNOS and COX-2 using a Western blotting assay. Figure 3 showed that the expression levels of iNOS and COX-2 were significantly elevated after LPS stimulation. However, the up-regulation of the cellular protein levels of iNOS and COX-2 were deceased by SCL pre-treatment in a concentration-dependent manner (Figure 3A–C).

### 2.4. SCL Repressed the Production of Cytokines and Chemokines in LPS-Stimulated RAW264.7 Cells

**Figure 4:**
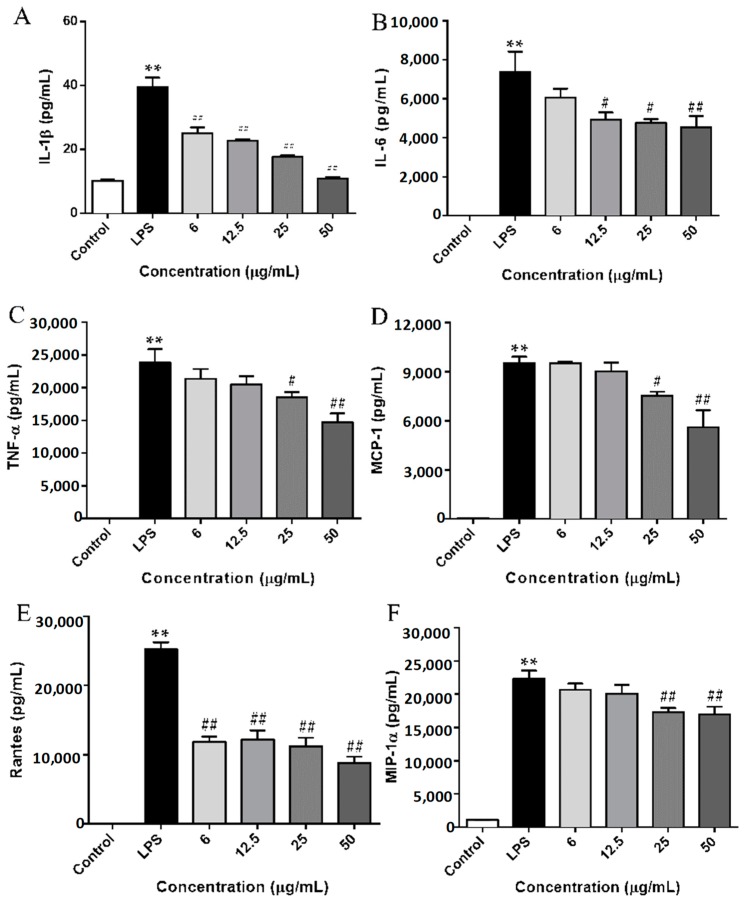
Effect of SCL on the production of cytokines and chemokines in LPS-stimulated RAW264.7 cells. The cells were plated in 24-wells and incubate for 12 h, and then the macrophages were pre-treated with different concentrations of SCL for 1 h and then stimulated with or without LPS (1 μg/mL) for 24 h. IL-1β (**A**), IL-6 (**B**), TNF-α (**C**), MCP-1 (**D**), Rantes (**E**), and MIP-1α (**F**) concentrations in the cell culture medium were measured by ELISA. Values given are the mean ± SEM of 4 independent experiments; ** *p* < 0.01 vs. unstimulated cells. **^#^** *p* < 0.05, **^##^** *p* < 0.01 vs. LPS-stimulated cells using a one-way ANOVA followed by Dunnett’s multiple comparisons test.

To further examine the inhibitory effect of SCL on LPS-induced inflammatory responses, we measured pro-inflammatory cytokines (IL-1β, IL-6, and TNF-α) and chemokines (MCP-1, Rantes, and MIP-1α) released into the culture medium using an ELISA assay. As shown in Figure 4A–C, 24 h stimulation with LPS induced obvious elevation levels of cytokines including IL-1β, IL-6, and TNF-α in cell culture medium. However, the elevated levels of these cytokines were significantly inhibited by LPS treatment in a dose-dependent manner. Moreover, the production of TLR4-mediated chemokines, such as MCP-1, Rantes, and MIP-1α, were remarkably increased after LPS stimulation. SCL treatment repressed the release of MCP-1, Rantes, and MIP-1α in a concentration dependent manner (Figure 4D–F).

### 2.5. SCL Regulated on the Components of IKKα/β/NF-κB, MAPKs/AP-1 and TBK1/IRF3 Signaling Pathways

**Figure 5:**
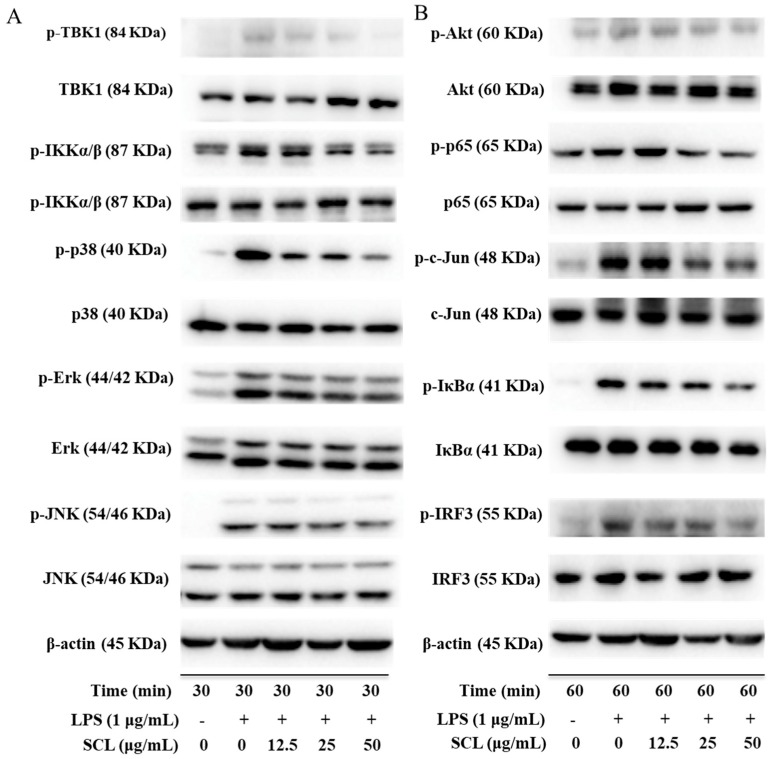
SCL affects the components of IKKα/β/NF-κB, MAPKs/AP-1 and TBK1/IRF3 pathways in LPS-stimulated RAW264.7 cells. Cells were treated with SCL at the indicated concentrations for 1 h and then stimulated with LPS for 30 min or 60 min. Total and phosphorylated forms of corresponding proteins were detected by Western blotting (**A**,**B**).

**Figure 6:**
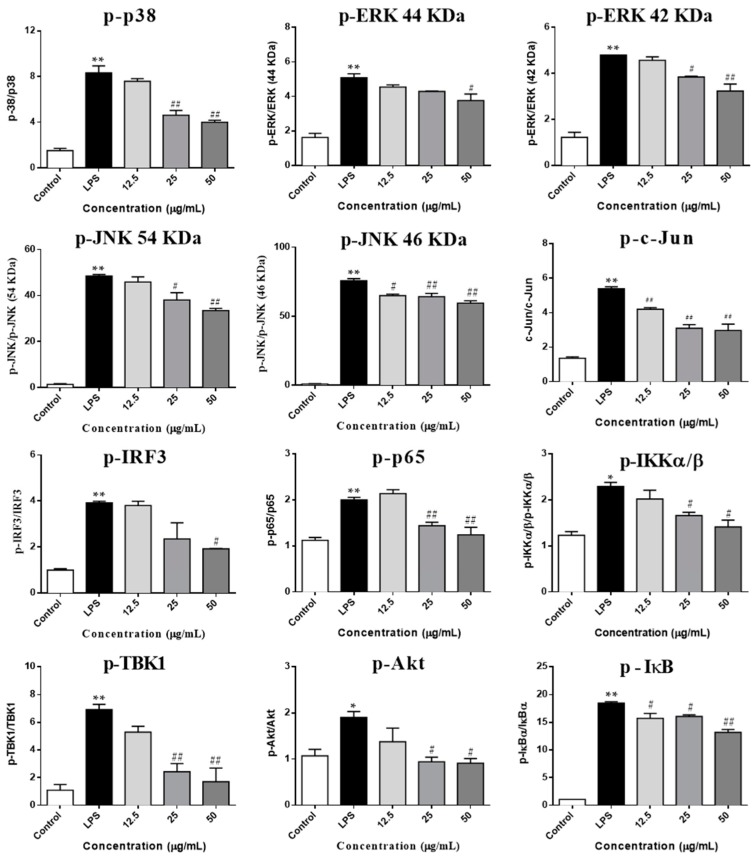
Effect of SCL on the components of IKKα/β/NF-κB, MAPKs/AP-1 and TBK1/IRF3 pathways in LPS-stimulated RAW264.7 cells. The data presented in bar charts indicates the ratio of p-IKKα/β/IKKα/β, p-IκBα/IκBα, p-TBK1/TBK1, p-p38/p38, p-ERK/ERK (44 KDa), p-ERK/ERK (42 KDa), p-JNK/JNK (54 KDa), p-JNK/JNK (46 KDa), p-p65/p65, p-c-Jun/c-Jun, p-IRF3/IRF3, and p-Akt/Akt in RAW264.7 cells. Values given are the mean ± SEM of 3 independent experiments; * *p* < 0.05, ** *p* < 0.01 vs. unstimulated cells. **^#^** *p* < 0.05, **^##^** *p* < 0.01 vs. LPS-stimulated cells using a one-way ANOVA followed by Dunnett’s multiple comparisons test.

The TLR4 cascade plays a pivotal role in the activation of macrophage cells and the involvement of Akt was also identified [24]. Therefore, to investigate whether SCL regulates these pathways, we used Western blots to examine the phosphorylation of three key transcription factors including NF-κB, AP-1 and IRF3 in LPS-stimulated RAW264.7 macrophages. We observed that the phosphorylation of NF-κB/p65, AP-1/c-Jun and IRF3 was significantly up-regulated after LPS stimulation. SCL treatment obviously decreased the phosphorylation of NF-κB/p65, AP-1/c-Jun and IRF3 in a concentration dependent manner. Moreover, some effectors of the TLR4 signaling pathways related upstream proteins were also determined. As shown in Figure 5 and Figure 6, the phosphorylation of IKKα/β and IκB was significantly elevated by LPS treatment, pre-treatment with SCL for 1 h markedly suppressed LPS-induced phosphorylation of IKKα/β and IκB. Moreover, after stimulation of RAW264.7 cells with LPS, the phosphorylation of three MAPKs subunits (p38, ERK and JNK) was obviously increased, indicating that LPS activated MAPKs signaling (Figure 5A and Figure 6). However, it was found that SCL treatment inhibited the phosphorylation of p38, ERK and JNK in a concentration dependent manner. In addition, the phosphorylation of NF-κB subunit p65 (ser536) and AP-1 subunit c-Jun (ser73) were significantly increased after 1 h LPS stimulation. SCL treatment concentration-dependently repressed the phosphorylation of p65 and c-Jun (Figure 5B and Figure 6). Moreover, Akt signaling also plays a role in LPS-triggered inflammatory response in macrophages. As shown in Figure 5B, the phosphorylation of Akt (ser473) was markedly increased after LPS stimulation in RAW264.7 macrophages, while SCL inhibited the phosphorylated Akt (ser473) in a concentration dependent manner (Figure 5B and Figure 6).

In addition, the involvement of the TBK1/IRF3 signaling pathway was also investigated in LPS stimulated RAW264.7 cells. Figure 5 and Figure 6 showed that the phosphorylation levels of TBK1 and IRF3 were obviously enhanced after LPS stimulation. However, SCL treatment concentration-dependently inhibited the phosphorylated the TBK1 and IRF3, suggesting that SCL also inhibited TBK1/IRF3 signaling pathway (Figure 5B and Figure 6).

### 2.6. SCL Suppressed the Nuclear Translocation of AP-1, IRF3, and NF-κB in RAW264.7 Cells

**Figure 7:**
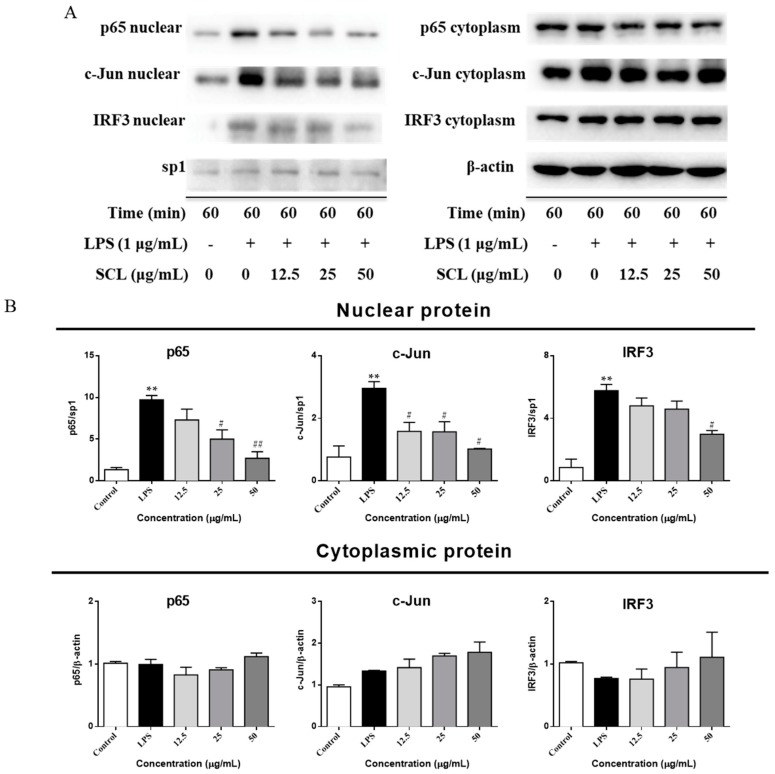
Effect of SCL on the nuclear and cytoplasmic protein levels of NF-κB, AP-1 and IRF3 in LPS-stimulated RAW264.7 cells. Cells were pre-treated with SCL in different concentrations as indicated for 1 h and then stimulated with LPS for 60 min. The cytoplasmic and nuclear protein levels of NF-κB (p65), AP-1 (c-Jun), and IRF3 were determined by Western blotting (**A**). Bar graphs show the relative levels of cytoplasmic and nuclear p65, IRF3, and c-Jun (**B**). Values given are the mean ± SEM of 3 independent experiments. ** *p* < 0.01 vs. unstimulated cells. **^#^** *p* < 0.05, **^##^** *p* <0.01 vs. LPS-stimulated cells using a one-way ANOVA followed by Dunnett’s multiple comparisons test.

**Figure 8:**
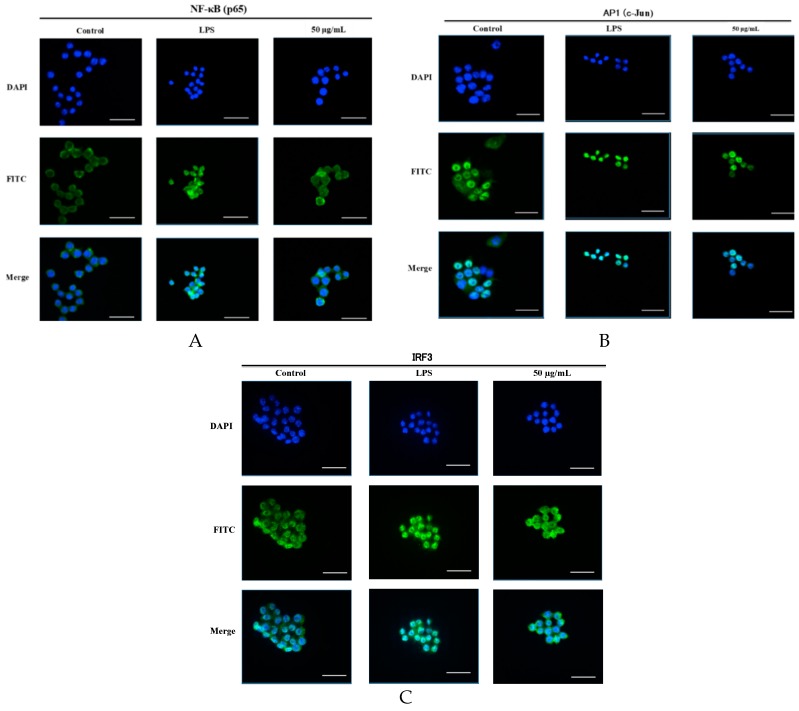
Effect of SCL on the nuclear translocation of NF-κB, AP-1 and IRF3 in LPS-stimulated RAW264.7 cells. Cells were pre-treated with SCL in different concentrations as indicated for 1 h and then stimulated with LPS for 60 min. The subcellular localization of NF-κB (p65), AP-1 (c-Jun), and IRF3 was determined using an immunofluorescence assay (**A**–**C**). The images of these three transcriptional factors were acquired by confocal microscope. The bar in each image represents 33 μm.

As shown in Figure 5, the nuclear protein levels of IRF3, NF-κB heterodimer component (p65), and one of the AP-1 components (c-Jun) were markedly increased after a 60 min LPS treatment. However, stimulation of cells with LPS in the presence of SCL resulted in the reduction of their nuclear protein levels. (Figure 7A,B). The cytosolic protein levels of these three transcription factors were not obviously changed after SCL treatment (Figure 7A,B). Furthermore, the translocation of these three transcription factors to the nucleus in RAW264.7 cells was analysed using immunofluorescence staining. The immunofluorescence images revealed that NF-κB (p65), AP-1 (c-Jun), and IRF3 were normally sequestered in the cytoplasm, and nuclear translocation of these three transcriptional factors was nearly not observed in the cells in the absence of LPS. However, the nuclear localizations of NF-κB (p65), AP-1 (c-Jun), and IRF3 in RAW264.7 cells were significantly induced after stimulation with LPS, which were remarkably reduced after pre-treating the cells with SCL (Figure 8A–C).

## 3. Discussion

Natural products probably represent an ideal source to explore safe and effective agents for the management of inflammatory disorders [25]. In our present study, we used a classical inflammatory cell model, called LPS-stimulated RAW264.7 cells, to investigate the suppressive effect of SCL on inflammatory mediators. Many lignan components isolated from *Schisandra Chinensis* Baill are known as antioxidants and for their hepatitis healing activities [26]. Some researchers also reported that lignans from *Schisandra Chinensis*, including Schisandrin A, Schisandrin B, Schisandrin C, and Schisandrin, inhibited the production of NO and cytokines in vitro [27]. In line with the previous studies, we found that SCL remarkably reduced the extent of inflammation in LPS-activated RAW264.7 macrophages and the underlying mechanism may contribute to its regulative effects of NF-κB, AP-1, and IRF3 and their up-stream proteins.

NO is a key pro-inflammatory mediator and can influence many aspects of the inflammatory cascade ranging from its own expression to the recruitment of leucocytes to the effected tissue [28]. It plays an important role in the pathogenesis of inflammatory disorders of the joint, gut and lungs [29]. PGE2 is a potent inflammatory mediator that is synthesized by various cell types, including endothelial cells, leukocytes and macrophages [30]. PGE2 modulates the classic signs of inflammation, including redness and painful swelling [31]. Therefore, inhibition of NO and PGE2 production represents important therapeutic advance in the management of inflammatory diseases. In addition, as NO is synthesized from L-arginine by iNOS [32] and PGE2 is produced from arachidonic acid metabolites by COX-2 [30]. We detected the production of NO and PGE2 and the expression of iNOS and COX-2 in LPS stimulated RAW264.7 cells. It was found that upon stimulation of the RAW264.7 cells by LPS, the release of the NO and PGE2 was markedly increased, and pre-treatment of RAW264.7 cells with non-toxic concentrations of SCL (6–50 μmol/L) reduced LPS-induced release of NO and PGE2. Moreover, SCL treatment obviously inhibited the expression of iNOS and COX-2 in LPS-stimulated RAW264.7 macrophages in a concentration dependent manner. These results suggested that the suppressive effect of SCL on NO and PGE2 production is the result of the inhibition of iNOS and COX-2 expression, respectively.

NF-κB, AP-1 and IRF3 are key transcription factors of macrophages and are required for the induction of many cytokines and chemokines genes, including those encoding TNF-α, IL-1β, IL-6, Rantes, MCP-1 and MIP-1α [33]. These inflammatory mediators can both directly engage in the induction of inflammation and act indirectly through promoting the differentiation of inflammatory immune cells [34]. Hence, anti-inflammatory agents inhibiting the release of these inflammatory cytokines and chemokines could hinder the inflammatory cascade response. Our data showed that SCL concentration dependently reduced LPS-induced release of cytokines (IL-1β, IL-6 and TNF-α) and chemokines (MCP-1, MIP-1α and Rantes) in RAW264.7 cells, suggesting that SCL has the potential ability to inhibit the production of inflammatory mediators.

After macrophages are stimulated with LPS, NF-κB, AP-1 and IRF3 are liberated and rapidly translocated into the nucleus, wherein they activate the transcription of their target cytokine and chemokine genes [34]. Our results showed that SCL effectively prevent the nuclear translocation of NF-κB (p65), AP-1 (c-Jun), and IRF3 in LPS-stimulated RAW264.7 cells. We also found that SCL treatment concentration dependently reduced the phosphorylation of these three transcription factors. These results suggested that SCL possess strong inhibitory effects on NF-κB, AP-1 and IRF3 signaling pathways. Moreover, the activation and nuclear translocation of NF-κB are required for its inhibitor (IκB) phosphorylation. The upstream protein of IκB is the IKK complex (consisting of IKKα, IKKβ, and IKKγ subunits), which in turn phosphorylates IκBα, leading to ubiquitin-dependent IκBα degradation and NF-κB activation [35]. Therefore, IKK-IκBα-NF-κB is a good targeting axis for alleviating inflammation. Our current study indicated that the phosphorylation of IκBα and IKKα/β was significantly elevated after LPS stimuli, and SCL treatment inhibited the phosphorylation of IκBα and IKKα/β in a concentration dependent manner, indicating that SCL may inhibit IKKα/β/IκBα/NF-κB axis. Moreover, MAPKs (consisting of JNK, ERK and p38) are also responsible for the regulation of many genes involved in the production of inflammatory mediators [36]. In response to LPS stimulation, three MAPKs are activated via phosphorylation of both tyrosine and threonine residues, and this phosphorylation leads to the activation of AP-1 [37]. Our data indicated that SCL treatment significantly suppressed the phosphorylation of p38, ERK, and JNK, showing that SCL effectively blocked the signal transduction by MAPK molecules. In addition, as the downstream protein of PI3K, Akt plays an important role in LPS-induced activation of the NF-κB signaling pathway [38]. It has been reported that the activated Akt phosphorylates IKK complex and promotes the nuclear translocation of NF-κB and leading to inflammatory genes expression [24]. In our present study, we observed that the phosphorylation of Akt was markedly elevated after LPS stimulation. However, SCL treatment inhibited the phosphorylation of Akt in a concentration dependent manner, suggesting that SCL treatment suppresses the Akt signaling.

Additionally, LPS stimulation also activates TBK1, which then phosphorylates the transcription factor IRF3 and, thereby, induce IRF3 dimerization, leading to transcriptional induction of inflammatory mediators, such as Rantes, IFN-α and IFN-β [39,40,41]. Consistent with previous studies, our research found that the phosphorylation of TBK1 was significantly up-regulated after LPS stimulation, and SCL treatment down-regulated the phosphorylated TBK1, indicating SCL treatment also inhibits the TBK1/IRF3 signaling pathway.

## 4. Materials and Methods

### 4.1. Reagents

Bovine serum albumin, 3-(4,5-dimethylthiazol-2-yl)-2,5-diphenylthiazolium bromide (MTT), lipopolysaccharide (LPS, Escherichia coli 055:B5) and Griess reagent (modified) were purchased from Sigma Chemical Co (St. Louis, MO, USA). Foetal bovine serum (FBS) was bought from Biological Industries (Beth-Haemek, Israel). Dulbecco’s Modified Eagle Medium (DMEM) was obtained from Corning Cellgro (Manassas, VA, USA). Penicillin-streptomycin solution was bought from Caisson labs (Smithfield, UT, USA). RAW264.7 cells, a BALB/c-derived murine macrophage cell line (ATCC no. TIB-71) was purchased from ATCC (Rockville, MD, USA). Interleukin 1β (IL-1β), interleukin-6 (IL-6), tumour necrosis factor α (TNF-α), monocyte chemoattractant protein (MCP)-1, macrophage inflammatory protein (MIP)-1α and regulated on activation normal T expressed and secreted (Rantes) protein ELISA kits were obtained from Thermo fisher scientific (San Diego, CA, USA). Prostaglandin E2 (PGE2) ELISA kit was obtained from Enzo life science (Exeter, UK). IκB kinase α/β (IKKα/β) and sp1 monoclonal antibodies were obtained from Santa Cruz Biotechnology (Santa Cruz, CA, USA). Interferon regulatory factor 3 (IRF3) anti-body was provided by Abcam (Cambridge, UK). NF-κB p65, phospho-NF-κB p65 (Ser536), Akt, phospho-Akt (Ser473), c-Jun, phospho-c-Jun (Ser73), cyclooxygenase-2 (COX-2), inducible nitric oxide synthase (iNOS), phospho-IRF3 (Ser396), IκBα, phospho-IκBα (Ser32), phospho-IKKα/β (Ser176/180), extracellular signal-regulated kinase (ERK), phospho-ERK (Thr202/Tyr204), c-Jun *N*-terminal kinase (JNK), phospho-JNK (Thr183/Tyr185), p38 mitogen-activated protein kinase (p38), phospho-p38 (Thr180/Tyr182), phosphoinositide (PI) 3-kinase p85, phospho-PI3 Kinse p85 (Tyr458)/p55 (Tyr199), TANK-binding kinase 1 (TBK1), phospho-TBK1 (Ser172), anti-rabbit IgG HRP linked anti-body and Alexa Fluor 488-conjugated secondary antibody were obtained from Cell signaling technology (Boston, MA, USA). Schisandrin, Schisandrol B, Schisantherin A, Schisandrin A, Schisandrin B and Schisandrin C were purchased from Dasf Biocompany (Nanjing, China), the purity of each lignan was higher than 98%.

### 4.2. Preparation of SCL

Schisandra crude extract (SCE) powder (the content of total lignans is 10%) was purchased from Nuoz Biocompany (Hunan, China). 550 g of SCE was mixed with 16.5 L of water, which contained 82.5 g of Tween 80 (Sigma-Aldrich Co., Darmstadt, Germany). Subsequently, ultrasound assist extraction of this mixture was performed for 3 times, and 30 min for each time. The water extract was then filtered and merged for further purification by using macroporous resin column chromatography. Briefly, the water extract was loaded onto a chromatographic column, which was packed with macroporous resin (SP207, Mitsubishi, Japan), and the diameter to the height of this column was 1:5. After the sample solution loading, water was used to remove the aqueous impurities and then eluted with 5 column volumes of 20% and 90% methanol aqueous solution, respectively. Eluates were collected and evaporated under reduced pressure using a rotator evaporator to afford a residue, which was then dissolved by methanol. The solution was added into a silica sphere C18 column, and then eluted by different concentrations of acetonitrile aqueous solutions (30%, 60% and 90%) at a flow rate of 1 mL/min, respectively. For each concentration, 3 column volumes of acetonitrile aqueous solution were consumed. The eluates were collected, merged, and evaporated under reduced pressure using a rotator evaporator. Finally, 11.1 g of SCL was obtained and identified by high-performance liquid chromatography (HPLC).

### 4.3. Characterization of SCL

To control the quality of the SCL, HPLC analysis was conducted using an Agilent 1100 system (Agilent Technologies Inc, La Jolla, CA, USA) consisted of a quaternary pump, a standard auto-sampler, a column oven and a diode array detector. Chromatographic separation was performed on an Alltech chrome C18 column (4.6 × 250 mm^2^, 5 μm). A linear gradient system consisted of A (acetonitrile) and B (water). The gradient elution profile was as follows: 0–30 min, 43% A; 30–32 min, 43–46%A; 32–53 min, 46–50% A; 53–64 min, 50–62% A; 64–83 min, 62% A; 83–93 min, 62–90% A; 93–98 min, 90–100% A. The flow rate was 1.0 mL/min, with the column temperature maintained at 35 °C and the injection volume of samples was 10 μL in each experiment.

### 4.4. Cell Culture

The murine macrophage cell line, RAW264.7, was obtained from the American Type Culture Collection (Manassa, VA, USA) and maintained in a DMEM medium containing 10% heat-inactivated foetal bovine serum (FBS) and 1% antibiotics penicillin/streptomycin at 37 °C under 5% CO_2_. To prepare the sample solution for assays, SCL was freshly dissolved in DMSO, and then diluted with DMEM to a series of concentrations.

### 4.5. Cell Viability Assay

The effect of SCL on the viability of RAW264.7 cells was determined using an MTT assay. Briefly, RAW264.7 cells were seeded into 96-well plates at a density of 6000 cells/well. After 24 h incubation, SCL was added to the cells and incubated for 1 h, and then cells were treated with or without LPS (1 μg/mL) for 24 h at 37 °C and 5% CO_2_. After that, the medium was discarded and the cells were incubated with the MTT solution (0.5 mg/mL) for another 3 h. The supernatant was removed and the remaining formazan crystals were dissolved in 100 μL DMSO. The optical density was measured at 570 nm using a microplate spectrophotometer (BMG SPECTROstar Nano, Offenburg, Germany).

### 4.6. Detection of Nitric Oxide (NO)

The RAW264.7 cells were seeded at 2 × 10^5^ cells/mL on 24-well culture plates for 16 h. Various concentrations of SCL were prepared. After 1 h pre-treatment with indicated concentrations of SCL, the cells were then treated with or without LPS (1 μg/mL) for another 24 h. NO production was determined by measuring the accumulated nitrite (a stable degradation product of NO) in the culture medium with Griess reagent [42]. Absorbance at 540 nm was measured with a NaNO_2_ standard curve, and nitrite production was determined.

### 4.7. Enzyme-Linked Immunosorbent Assay (ELISA)

The production of IL-1β, IL-6, TNF-α, PGE2, MCP-1, MIP-1α and Rantes was determined by ELISA as previously described [43]. Briefly, RAW264.7 macrophages were seeded on 24-well culture plates at 2 × 10^5^ cells/well overnight. The cells were treated with SCL at 6–50 μg/mL for 1 h and then at the presence or absence of LPS (1 μg/mL) for another 24 h. Cell-free supernatants were collected for determination of cytokine and chemokine concentrations according to the manufacturer’s instructions.

### 4.8. Western Blot Analysis

Western blots wre conducted as previously described [33]. Briefly, RAW264.7 cells were seeded in 60 mm-diameter culture dishes (4 × 10^5^ cells) and treated with SCL at 12.5–50 μg/mL LPS (1 μg/mL) was added 1 h after SCL pre-treatment and the cells were incubated at 37 °C for 30 min or 60 min. The cells were then washed twice in ice-cold PBS, and then treated with ice-cold RIPA protein extraction buffer (Beyotime biotechnology, Beijing, China) containing 1% protease and phosphatase inhibitors. The cytoplasmic and nuclear proteins were prepared using nuclear extraction kit (Solarbio, Beijing, China) according to the manufacturer’s instructions. Equal amounts of protein were subjected to 10% SDS-polyacrylamide gel electrophoresis (PAGE) and then electro-transferred to polyvinylidene fluoride (PVDF) membranes (0.45 μm). After blocking with 5% non-fat milk, membranes were probed with indicated primary antibodies (1:1000) over night at 4 °C and were then blotted with anti-rabbit secondary anti-body. Visualization was performed using a Tanon 5200 Multi chemiluminescent imaging system (Tanon Science & Technology Co., Ltd., Shanghai, China) with enhanced-chemiluminescence substrate, and the blots were analysed using Image J software (National Institutes of Health [NIH], Bethesda, MD, USA). Protein levels wre normalized to the matching densitometric value of the internal control β-actin.

### 4.9. Immunofluorescence Staining

RAW264.7 cells were directly cultured on chamber slides (Thermo Scientific, Waltham, MA, USA) overnight to detect NF-κB/p65, AP-1/c-Jun, and IRF3 localization by immunofluorescence assays. Briefly, after 60 min of stimulation with LPS (1 μg/mL) in the presence or absence of SCL, the cells were washed with cold PBS, and fixed with 4% formaldehyde in PBS for 15 min at room temperature. Subsequently, the cells were permeabilized with 0.25% Triton-x for 30 min at 37 °C and followed by 10 min at room temperature. After blocking the cells with 2% BSA for 1 h, primary antibodies against NF-κB/p65 (1:300), c-Jun (1:300), and IRF3 (1:100), respectively, were incubated overnight at 4 °C. After washing with PBS for 3 times, cells were incubated for 1 h at room temperature with Alexa Fluor 488-conjugated secondary antibody (1:500). After the nuclei were stained with DAPI, and fluorescence was visualized using a Nikon A1R Eclipse Ti confocal microscope (Nikon Corp., Tokyo, Japan).

### 4.10. Statistical Analysis

Data were presented as the mean ± SEM of at least three triplicate determinations. Statistical differences were determined using one-way ANOVA followed by Dunnett’s multiple comparisons test. GraphPad Prism 5.0 (GraphPad Software, San Diego, CA, USA) was used for statistical analyses. Differences were considered significant at *p* < 0.05.

## 5. Conclusions

**Figure 9:**
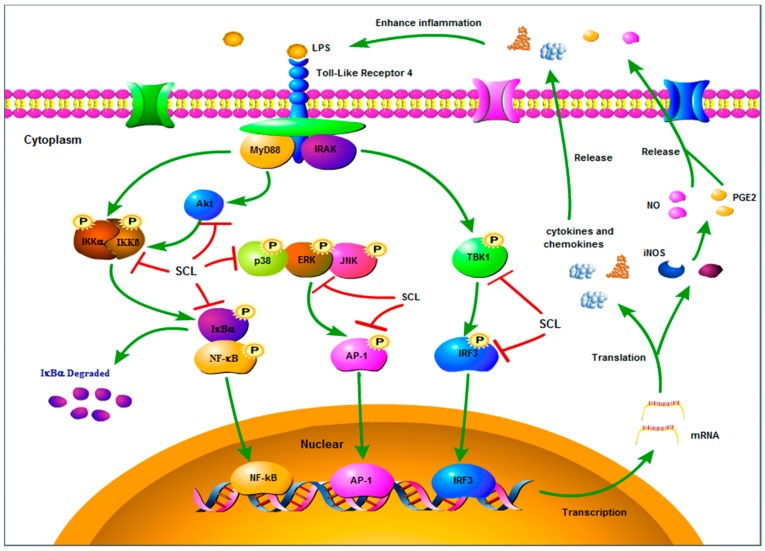
Proposed molecular mechanisms underlying the inhibitory effects of SCL on the production of inflammatory mediators. SCL inhibits IKKα/β/NF-κB, MAPKs/AP-1 and TBK1/IRF3 pathways in LPS-stimulated RAW264.7 cells.

In summary, the current study showed that SCL possesses inhibitory effect on inflammatory mediators including NO and PGE2 secretion and the expressions of iNOS and COX-2. Moreover, SCL treatment markedly reduced the production of pro-inflammatory cytokines and chemokines. Mechanistic investigation showed that inhibition of IKKα/β/NF-κB, MAPKs/AP-1, and TBK1/IRF3 pathways were associated with the suppressive effect of SCL on inflammatory mediators in LPS-stimulated RAW264.7 cells (Figure 9). The results provide further pharmacological basis for the clinical application of SCL in the treatment of inflammatory disorders and suggest that SCL is a promising modern agent for the intervention of inflammatory disorders, especially for LPS-associated inflammatory diseases. Further studies will be conducted to validate the anti-inflammatory effect of SCL in an acute hepatitis mouse model.

## Figures and Tables

**Table 1:** The mean contents of each lignans.

Peak No.	Retention Time (min)	Compounds	Content in Sample (mg/g)
1	23.554	Schisandrin	303.03
2	33.907	Schisandrol B	89.86
3	64.140	Schisantherin A	44.93
4	81.137	Schisandrin A	57.47
5	90.831	Schisandrin B	103.45
6	93.303	Schisandrin C	7.84
